# P-1861. The Impact of Medication Assisted Therapy (MAT team) on Patients Enrolled in an OPAT Program

**DOI:** 10.1093/ofid/ofaf695.2030

**Published:** 2026-01-11

**Authors:** Jude Meniru, Margaret Williams, Drew Logan, Joseph Asteriou, Ashley Lipps, Mohammad Madhee Sobhanie

**Affiliations:** Division of Infectious Disease, Department of Internal Medicine, The Ohio State University Wexner Medical Center, Columbus, OH, USA, Columbus, OH; The Ohio State University Wexner Medical Center, Columbus, Ohio; 2. Division of Hospital Medicine, Department of Internal Medicine, The Ohio State University Wexner Medical Center, Columbus, OH, USA, Columbus, Ohio; 2. Division of Hospital Medicine, Department of Internal Medicine, The Ohio State University Wexner Medical Center, Columbus, OH, USA, Columbus, Ohio; The Ohio State University Wexner Medical Center, Columbus, Ohio; The Ohio State University, Columbus, Ohio

## Abstract

**Background:**

Outpatient parenteral antibiotic therapy (OPAT) is a method of treatment that allows patients who would require inpatient admission for the duration of their IV therapy to receive it in the outpatient setting. Persons who use or inject drugs (PWUD/PWID) are not always offered OPAT due to concern for tampering of lines, nonadherence, and elopement. Implementation of addiction medicine consults has demonstrated reduction in readmission rates for PWUD. Medication-assisted treatment (MAT) has been shown to aid in completion of OPAT in case reports but data is lacking for this purpose. The goal of our study is to evaluate the OPAT completion rate between patients who accepted MAT versus those who did not accept MAT.

**Figure 1:**
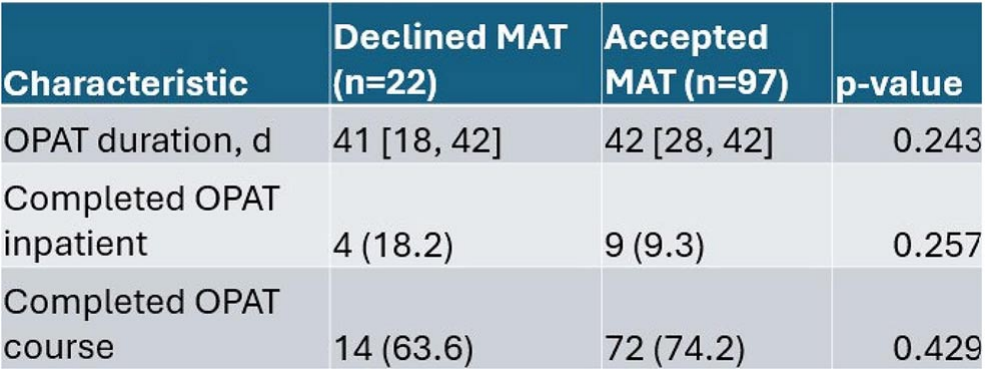
Overall completion of OPAT OPAT, Outpatient parenteral antibiotic therapy; MAT, Medication Assisted Treatment; n, Number of patients in category

**Figure 2:**
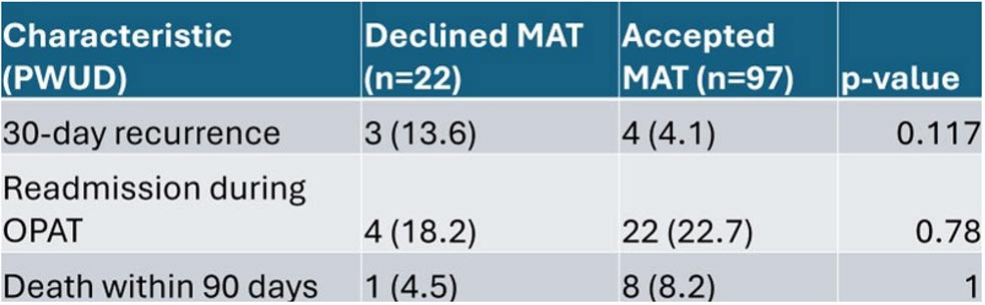
Overall Primary and Secondary Endpoints PWUD, Patients who use drugs, OPAT, Outpatient parenteral antibiotic therapy; MAT, Medication Assisted Treatment; n, Number of patients in category

**Methods:**

This is a single-center retrospective cohort study comparing OPAT completion rates, readmission within OPAT period, and 30-day recurrence of infection for patients requiring OPAT for invasive bacterial infections from July 1, 2023 to June 30, 2024 who either accepted or declined MAT during hospitalization. Exclusion criteria included patients younger than 18, prisoners, and encounters that did not include both an OPAT and Addiction Medicine consult and MAT. Categorical variables were reported as counts and percentages using the Fisher exact test for comparison. Continuous variables were reported as medians and interquartile ranges (IQRs) from the 25th to the 75th percentiles and compared using the t test. A P value of < 0.05 was considered significant.

**Figure 3:**
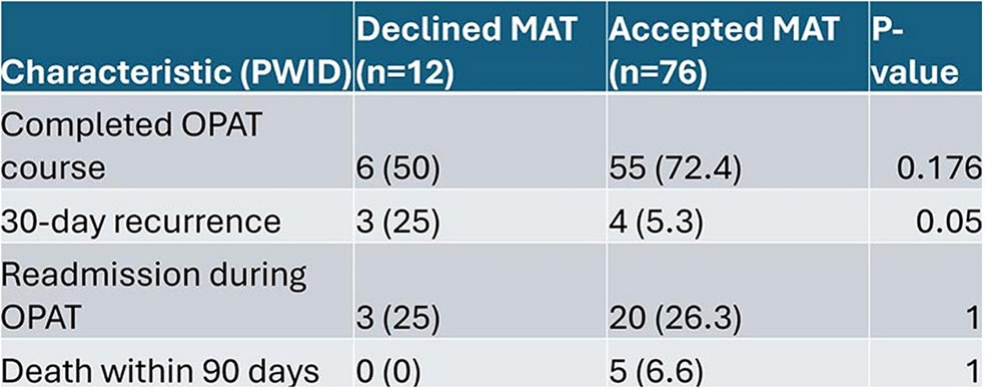
PWID Primary and Secondary Endpoints PWID, Patients who inject drugs; OPAT, Outpatient parenteral antibiotic therapy; MAT, Medication Assisted Treatment; n, Number of patients in category

**Results:**

236 patients were identified as having had an OPAT and being offered MAT, and 117 were excluded based on criteria. Of the 119 patients included, 97 accepted MAT and 22 declined. There was not a significant difference in these measured outcomes between PWUD who either accepted or declined MAT during hospitalization. Subgroup analysis did show a significant reduction in recurrence of infection for PWID who accepted MAT (5.3% vs 25%).

**Conclusion:**

We did not find a difference in patients who accepted MAT versus those who did not accept MAT and OPAT completion rates, however we did find a statistically significant decrease in 30-day recurrence of infection in the IVDU subgroup population. More studies are needed to better identify what other factors contribute to successful completion of OPAT therapy in patients who inject drugs.

**Disclosures:**

All Authors: No reported disclosures

